# Emerging Link between Alzheimer's Disease and Homeostatic Synaptic Plasticity

**DOI:** 10.1155/2016/7969272

**Published:** 2016-02-25

**Authors:** Sung-Soo Jang, Hee Jung Chung

**Affiliations:** ^1^Department of Molecular and Integrative Physiology, University of Illinois at Urbana-Champaign, Urbana, IL 61801, USA; ^2^Neuroscience Program, University of Illinois at Urbana-Champaign, Urbana, IL 61801, USA

## Abstract

Alzheimer's disease (AD) is an irreversible brain disorder characterized by progressive cognitive decline and neurodegeneration of brain regions that are crucial for learning and memory. Although intracellular neurofibrillary tangles and extracellular senile plaques, composed of insoluble amyloid-*β* (A*β*) peptides, have been the hallmarks of postmortem AD brains, memory impairment in early AD correlates better with pathological accumulation of soluble A*β* oligomers and persistent weakening of excitatory synaptic strength, which is demonstrated by inhibition of long-term potentiation, enhancement of long-term depression, and loss of synapses. However, current, approved interventions aiming to reduce A*β* levels have failed to retard disease progression; this has led to a pressing need to identify and target alternative pathogenic mechanisms of AD. Recently, it has been suggested that the disruption of Hebbian synaptic plasticity in AD is due to aberrant metaplasticity, which is a form of homeostatic plasticity that tunes the magnitude and direction of future synaptic plasticity based on previous neuronal or synaptic activity. This review examines emerging evidence for aberrant metaplasticity in AD. Putative mechanisms underlying aberrant metaplasticity in AD will also be discussed. We hope this review inspires future studies to test the extent to which these mechanisms contribute to the etiology of AD and offer therapeutic targets.

## 1. Introduction

Neurons communicate with each other at specialized intercellular junctions, called synapses. The strength of synaptic transmission can be dynamically and persistently altered in response to changes in neuronal activity. In the book* The Organization of Behavior*, Donald Hebb postulated that connections between neurons that are simultaneously active are strengthened [1]. Such “Hebbian plasticity” was first demonstrated at excitatory glutamatergic synapses in rabbit hippocampus by the seminal work of Bliss and Lomo [2]. High frequency stimulation of presynaptic axons in the perforant pathway induces stronger and long-lasting excitatory postsynaptic potentials (EPSPs) in neurons of the postsynaptic dentate gyrus [2]. This long-term potentiation (LTP) of excitatory synaptic strength lasts hours to months [2] and can be induced electrically in brain slices as well as* in vivo* in behaving animals [2, 3]. Hence, the associative and input-specific synaptic plasticity such as LTP and its counterpart long-term depression (LTD) is thought to underlie cellular correlates of learning and memory [4–7].

Hebbian plasticity also represents a positive feedback mechanism. Once LTP is induced, saturated synapses undergo further potentiation with greater ease than before the LTP induction, leading to unstable runaway excitation [8–10]. Similarly, continuous synaptic depression during LTD could result in unnecessary synaptic silencing and elimination [8–10]. In order to sense and counteract destabilizing effects of LTP and LTD, neurons employ negative feedback processes called homeostatic synaptic plasticity [11–14]. This adaptive plasticity offers a compensatory refinement of synaptic strength to maintain the stability of network activity within a physiologic limit [13–15]. For example, prolonged elevation of neuronal activity results in compensatory downscaling of synaptic strength to prevent hyperexcitation, whereas prolonged suppression of neuronal activity leads to compensatory upscaling of synaptic strength to prevent synapse silencing and elimination [13–15]. Without this homeostatic mechanism, the capacity of an active synapse would get saturated due to unconstrained potentiation, limiting its ability to store information (i.e., memory). Homeostatic synaptic plasticity is therefore a vital partner of Hebbian synaptic plasticity.

Defects in homeostatic synaptic plasticity could, in principle, cause abnormal Hebbian plasticity at synapses, leading to pathologic levels of synaptic potentiation or elimination in neurologic diseases. For example, Alzheimer's disease (AD) is characterized by progressive and irreversible memory impairment [16] and associated with inhibition of LTP and enhancement of LTD in the hippocampus [17–27]. While physiologic levels of soluble amyloid-*β* (A*β*) oligomers have been shown to enhance synaptic activity and LTP [28, 29], pathologic levels of soluble A*β* oligomers impair LTP and enhance LTD in acute hippocampal slices [30–33]. Such impairment in Hebbian synaptic plasticity correlates strongly with memory impairment in early AD when A*β* plaques and neuronal degeneration are minimal [34–36]. Recent studies suggest that this abnormal Hebbian plasticity is due to pathologic engagement or disruption of metaplasticity [27, 32, 37], a form of homeostatic synaptic plasticity that controls the induction threshold of LTP and LTD [38]. Interestingly, A*β*-induced aberrant hyperexcitability is found in cortical and hippocampal neuronal networks of human AD and mouse models of AD [39–45]. Further, epileptiform electrical seizures and neuronal activity stimulate A*β* synthesis and its release from the neurons in the hippocampus [46–48]. Indeed, a pathologic positive feedback loop between A*β* production and neuronal hyperexcitability would favor LTP inhibition and LTD induction. 

These studies have provided a possible link between abnormal metaplasticity and cognitive dysfunction in AD pathogenesis, although our knowledge on the underlying mechanisms is limited. An understanding of the molecular mechanisms through which altered metaplasticity contributes to AD synaptopathology will be crucial in decoding the etiology of AD and may facilitate “correcting metaplasticity” as a putative novel therapy to restore Hebbian synaptic plasticity and treat cognitive dysfunction in early AD. In this paper, we review recent studies demonstrating aberrant metaplasticity in AD and discuss the possible underlying mechanisms focused on glutamate receptor regulation.

## 2. Abnormal Hebbian Synaptic Plasticity in AD

AD is a neurodegenerative disorder characterized by progressive and irreversible cognitive decline [16]. It is the 6th leading cause of death in the United States and the most common cause of dementia, which affects over 44 million people worldwide [49]. The molecular hallmarks of AD are amyloid plaques (extracellular deposits consisting of aggregated insoluble A*β*) and neurofibrillary tangles (intracellular filamentous aggregates of hyperphosphorylated tau) in the hippocampus and cortices [50–52], the brain regions critical for learning and memory. Interestingly, genetic suppression of endogenous tau blocks cognitive dysfunction in AD animal models, in which A*β* expression has been increased using a transgene [53–55], suggesting that tau acts downstream of A*β* in AD pathogenesis. 

Importantly, soluble A*β* peptides rather than insoluble amyloid plaques have emerged to play critical roles in the early stages of AD pathogenesis. First, amyloid plaques are found at later stages after memory loss is already evident in humans and AD animal models with genetically elevated A*β* level [17–27]. Second, intracranial injection of soluble A*β* oligomers is sufficient to cause memory loss [29, 33, 56–59]. Third, rare early-onset autosomal dominant familial AD (FAD) is associated with increased levels of soluble A*β* due to mutations in genes whose protein products are involved in A*β* production and processing [60, 61]. A*β* peptides are generated by successive proteolysis of amyloid-*β* precursor protein (APP), a large transmembrane glycoprotein that is initially cleaved by the *β*-site APP-cleaving enzyme 1 (BACE1) and subsequently by *γ*-secretase in the transmembrane domain [62–64]. The FAD mutations are found in APP and presenilins [60, 61], which are catalytic components of *γ*-secretase [65]. Lastly, a major genetic risk factor for most AD (i.e., sporadic AD) is polymorphic *ε*4 allele of apolipoprotein E [66, 67]. The encoded ApoE4 is less efficient in clearing A*β* than the common ApoE3, suggesting a strong association between sporadic AD and increased levels of soluble A*β* [68].

How could pathologic levels of soluble A*β* oligomers cause cognitive dysfunction? The first clue came from the studies in AD mouse models with genetically elevated A*β* [17–27]. Before the development of amyloid plaques is evident, these AD mouse models display severe impairment of hippocampal LTP [17–27]. Furthermore, LTD is induced in these AD mouse hippocampi with subthreshold stimulations, which normally cannot induce LTD in wild-type control mice [17–27]. Subsequent studies have shown that direct application of soluble A*β* oligomers (synthetic, cell-culture secreted, or AD brain-derived) at pathologic levels inhibits LTP and enhances LTD in acute hippocampal slice [30–33]. A persistent and unchecked decrease in synaptic strength is expected to lead to the pathologic elimination of synapses [69–71]. Indeed, decreases in synapse density are evident in hippocampi of patients with early AD [72–75]. Therefore, abnormal Hebbian synaptic plasticity is thought to be the basis of memory loss in early AD when amyloid plaques and neuronal degeneration are minimal [34–36]. 

## 3. Is Abnormal Hebbian Synaptic Plasticity due to Defective Homeostatic Synaptic Plasticity in AD?

**Figure 1:**
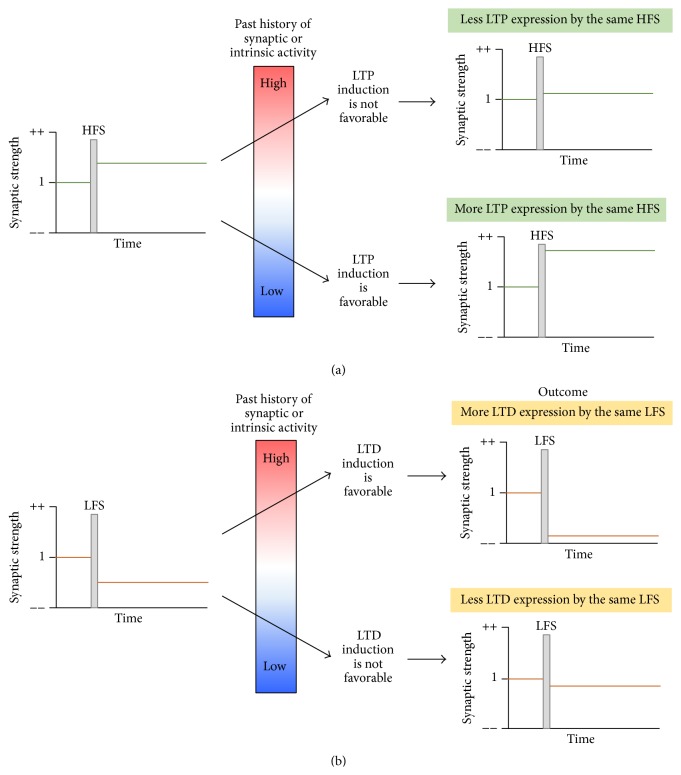
Metaplasticity. The induction threshold of LTP and LTD can be modified as a consequence of overall past synaptic or intrinsic activity of postsynaptic neurons. Such compensatory adjustment called “metaplasticity” provides stability to neuronal networks that support Hebbian synaptic plasticity. (a) LTP induction by conventional high frequency stimulation (HFS) is favorable in the neurons whose previous synaptic and intrinsic activities were low. (b) LTD induction by conventional low frequency stimulation (LFS) is favorable in the neurons whose previous synaptic and intrinsic activities were high.

Decades of studies cited above have compared the magnitudes of LTP and LTD in AD transgenic mouse models to determine if pathologic levels of soluble A*β* oligomers affect Hebbian synaptic plasticity. However, the absolute changes in the LTP and LTD magnitudes vary with age and AD mouse model [147], suggesting age- and strain-dependent differences for the induction threshold of LTP and LTD in these animals. The induction thresholds of LTP and LTD can be modified as a consequence of previous postsynaptic neuronal activity (Figure 1) [10, 148, 149]. LTP induction is favorable in neurons whose previous synaptic and intrinsic activities were low, whereas LTD induction is preferred when the previous activities were high [10, 148, 149]. Such compensatory adjustment of the induction thresholds for LTP and LTD, called “metaplasticity,” occurs as a form of homeostatic synaptic plasticity and provides stability to neuronal networks and supports Hebbian synaptic plasticity [10].

Hence, it is possible that the abnormal Hebbian synaptic plasticity in AD could arise from the defects in metaplasticity. Several studies have provided supporting evidence for this hypothesis. Aberrant neuronal hyperexcitability has been observed in cortical and hippocampal neuronal networks of patients with early AD [150] and FAD AD mouse models with heightened levels of APP and A*β* [21, 41–44, 151]; this is also consistent with reports that patients with early AD and FAD animal models exhibit epileptic seizures [21, 45, 152–160]. Pharmacological inhibition of epileptic seizures inhibits memory loss in AD mouse models [156], implicating critical roles of aberrant neuronal hyperexcitability in cognitive dysfunction presented early in AD pathogenesis [39, 40]. Hence, A*β*-induced cognitive dysfunction in early AD may result from the inability of neurons to adapt to persistent increases in overall neural network activity rather than the absolute changes in LTP and LTD magnitudes.

Additional support for this hypothesis comes from the report that soluble A*β* oligomers result in excessive activation of N-methyl D-aspartate receptors (NMDARs) containing GluN2B subunits, causing LTP inhibition and LTD facilitation via ERK and CREB signaling pathways [161]. GluN2B-selective antagonists effectively prevent A*β*-induced LTP inhibition [161–163], suggesting that early activation of extrasynaptic NMDARs primes the synapse to inhibit LTP induction and facilitate LTD induction. Consistent with this notion, GluN2B-selective antagonists prevent priming-induced inhibition of LTP [164]. The beneficial effects of the partial NMDAR antagonist memantine in AD also support the possible role of metaplasticity in AD-associated synaptic dysfunction because memantine does not block LTP acutely but restores LTP induction impaired by tonic NMDAR activation [165, 166].

**Figure 2:**
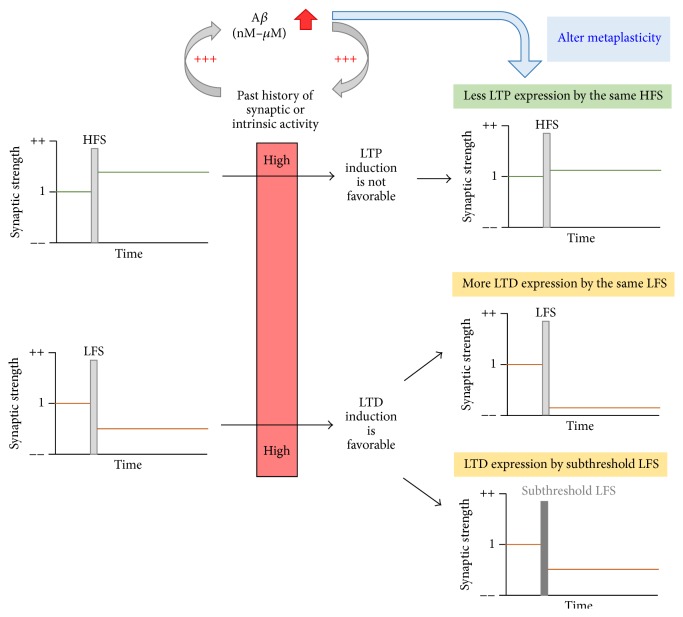
Aberrant metaplasticity in AD. A*β* increases the activity of excitatory neurons, which in turn stimulates synthesis and release of A*β* in a positive feedback loop, leading to pathologic accumulation of A*β*. Neuronal hyperexcitability or early activation of GluN2B-containing NMDAR by heightened A*β* expression induces aberrant metaplasticity, leading to inhibition of LTP by HFS and enhancement of LTD in the hippocampus by LFS or normal LTD induction by subthreshold LFS.

A recent study by Megill et al. has provided direct evidence for impaired metaplasticity in an AD transgenic mouse model [27]. This study examined frequency- and age-dependent synaptic plasticity in the APP/PS1 AD mouse model [27], which has two FAD-linked mutations (a Swedish mutation in APP and a deletion FAD mutation in exon 9 of presenilin-1) [167]. These mutations increase total A*β* production, resulting in a higher level of aggregation-prone A*β*42 peptides, and accelerated AD pathology [168] and age-dependent cognitive deficits [169, 170]. While the wild-type mice show a shift of the induction threshold to favor LTP and suppress LTD at the hippocampal CA1 Schaffer collateral synapses with age, the APP/PS1 transgenic mice fail to undergo this normal developmental metaplasticity [27]. As a result, the magnitudes of LTP and LTD remained the same in the APP/PS1 transgenic mice from when they were young (1 month of age) until they were adult (6 months of age). When the absolute magnitudes of LTP and LTD were compared, the adult APP/PS1 mice display LTP inhibition and LTD facilitation compared to age-matched wild-type mice [27]. Although electrophysiological characterization of other AD mouse models with elevated A*β* levels should be performed to see if impaired developmental metaplasticity is a general phenomenon for AD, these findings suggest that the Hebbian synaptic plasticity defects in AD could be due to the inability of neurons to undergo developmental metaplasticity (Figure 2).

## 4. Putative Mechanisms Underlying Defective Homeostatic Synaptic Plasticity in AD

How can pathologic levels of soluble A*β* oligomers cause aberrant metaplasticity in AD? One way to mediate metaplasticity is to alter the induction mechanisms of LTP and LTD by regulating the function of NMDARs [10, 171–174] because calcium (Ca^2+^) influx through NMDARs at the postsynaptic density (PSD) is critical for inductions of NMDAR-dependent LTP and LTD [175–177]. An effective means to alter Ca^2+^ current per unit charge through NMDAR is to change subunit composition of NMDAR [177]. Such a change influences Ca^2+^/calmodulin-dependent protein kinase II (CaMKII) interaction with NMDARs and has been shown to control Hebbian synaptic plasticity [178]. For example, GluN2B-containing NMDARs bind to CaMKII with high affinity whereas those containing GluN2A interact with CaMKII with low affinity. Consistent with their decreased affinity for CaMKII, altering synaptic NMDARs from GluN2B-containing receptors to GluN2A-containing ones markedly reduces LTP induction [178]. Developmental metaplasticity in the visual cortex has also been suggested to involve experience-dependent changes in the GluN2 subunit composition of NMDARs that influence the induction thresholds of LTP and LTD [173, 179].

Soluble A*β* oligomers have been shown to decrease glutamate reuptake and subsequently increase extracellular glutamate levels [32, 180]. Such glutamate spillover would activate extracellular NMDARs, which are mostly composed of GluN2B-containing NMDARs at mature synapses [175, 176]. Indeed, soluble A*β* oligomers enhance activation of GluN2B-containing NMDARs more rapidly than synaptic depression and such actions would prime excitatory synapses to inhibit LTP induction and favor LTD induction [161]. However, NMDAR subunit composition and current are similar between wild-type mice and APP/PS1 mice at all ages [27], suggesting that developmental metaplasticity defect in the APP/PS1 mice is not due to altered NMDAR function during the induction of LTP and LTD.

Another way to induce metaplasticity is to alter the expression mechanisms of LTP and LTD by regulating *α*-amino-3-hydroxy-5-methyl-4-isoxazolepropionic acid receptors (AMPARs) [181], which mediate the majority of excitatory synaptic current upon glutamate binding [182]. A major postsynaptic expression mechanism for LTP is the synaptic recruitment of AMPARs from a perisynaptic reserve pool and their subsequent stabilization at excitatory synapses, whereas that for LTD is the removal and internalization of synaptic AMPARs [175, 182]. Insertion and removal of synaptic AMPARs during the expression of LTP and LTD, respectively, are tightly regulated processes by phosphorylation of AMPAR subunit GluA1 at Ser-845 and Ser-831 [183–185]. Phosphorylation of GluA1 at Ser-845 by protein kinase A (PKA) is necessary for synaptic targeting of GluA1 driven by CaMKII [186], whereas dephosphorylation at Ser-845 mediates GluA1 internalization [183, 187, 188] and NMDAR-dependent LTD [185]. In addition, Ser845 phosphorylation of GluA1 mediates synaptic insertion of Ca^2+^-permeable GluA1-containing AMPARs during synaptic scaling in cultured dissociated cortical neurons upon chronic activity deprivation [92] and homeostatic synaptic scaling in the visual cortex upon sensory deprivation [189, 190]. Phosphorylation of GluA1 at Ser831 by protein kinase C (PKC) [191] and CaMKII [192, 193] increases following LTP induction [184, 194] and supports LTP expression [183–185]. Although GluA1 phosphorylation at Ser-845 and Ser-831 has been shown to reduce the induction threshold for LTP [195, 196], adult APP/PS1 mice display normal levels of GluA1 phosphorylation and perisynaptic AMPARs compared to those of wild-type mice [27]. Hence, the developmental metaplasticity defect in APP/PS1 mice is not due to insufficient AMPAR availability for synaptic insertion; rather, it is due to regulation of AMPAR trafficking by means other than GluA1 phosphorylation.

**Figure 3:**
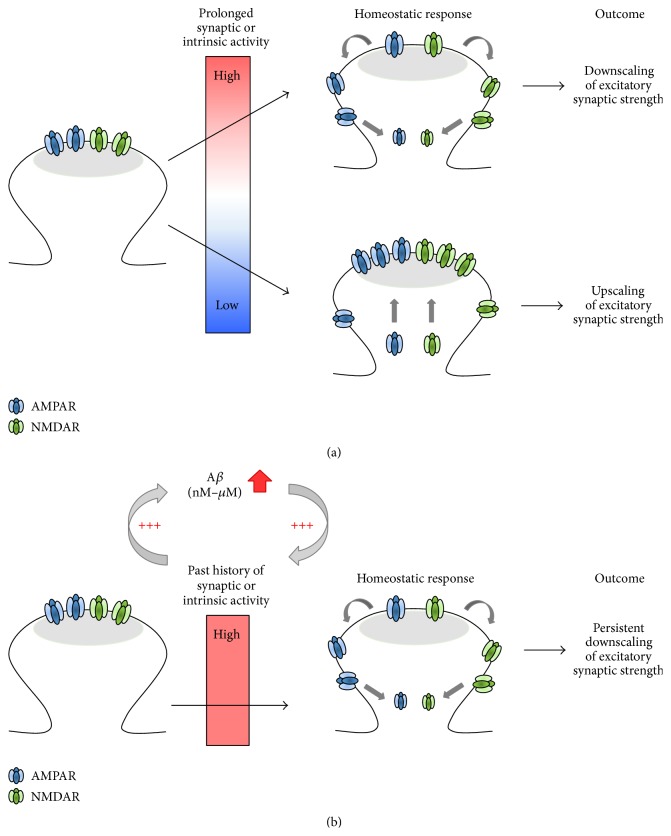
Postsynaptic expression mechanisms in normal and AD synapses. (a) In normal synapses, chronic activity blockade leads to synaptic scaling expressed by a compensatory increase in synaptic AMPAR density and current, whereas chronic activity elevation leads to synaptic downscaling expressed by a compensatory decrease in synaptic AMPAR density and current. (b) In AD, A*β* increases neuronal excitability and/or synaptic activity, leading to induction of synaptic downscaling. Because pathologic feedback loop continues to produce and release A*β*, synaptic downscaling becomes persistent and opposes the postsynaptic expression mechanisms for LTP.

Metaplasticity is a form of homeostatic synaptic plasticity in which the magnitude and polarity of synaptic plasticity are adjusted accordingly based on the past history of synaptic and neural activity [38]. Since metaplasticity can occur at a single synapse [197, 198], it is tempting to speculate that pathologic levels of A*β* may impair developmental metaplasticity by altering postsynaptic expression mechanisms of homeostatic synaptic plasticity (Figure 3). Homeostatic synaptic plasticity has been extensively investigated using primary dissociated culture of neocortical and hippocampal neurons (Table 1). In these studies, prolonged blockade of network activity for 48 hours (h) with the sodium channel blocker tetrodotoxin (TTX) induces a significant increase in AMPAR-mediated miniature excitatory postsynaptic current (mEPSC) amplitude and synaptic AMPAR density, indicating the postsynaptic expression of homeostatic synaptic scaling [78, 80, 94, 138, 199–201]. Conversely, mEPSC amplitudes are scaled down in dissociated neuronal culture after prolonged enhancement of network activity by KCl depolarization or blocking inhibitory neurotransmission with antagonists for A-type gamma-aminobutyric acid (GABA_A_) receptors, such as bicuculline [78, 80, 90, 199, 202, 203]. Interestingly, many of the crucial mediators of homeostatic synaptic plasticity have also been implicated in AMPAR regulation during LTP and LTD expression and AD pathology (Table 1). Taken together, these correlated functional roles raise an intriguing possibility that pathologic accumulation of A*β* may impair molecular mechanisms involved in homeostatic synaptic plasticity, which manifests as disruption of Hebbian synaptic plasticity in AD (Figure 3).

### 4.1. AMPAR Scaffolding Proteins

Glutamate receptor-interacting protein 1 (GRIP1) and PICK1 (protein interacting with C-kinase 1) are PDZ (postsynaptic density 95/discs large/zona occludens) domain-containing proteins that regulate AMPAR trafficking by binding to the same intracellular C-terminus of GluA2 [204, 205]. GRIP1 binding to the unphosphorylated GluA2 C-terminus promotes synaptic targeting of AMPARs [206] whereas PICK1 can bind to both phosphorylated and unphosphorylated GluA2 [206] and mediates activity-dependent endocytosis of GluA2-containing AMPARs and stabilizes them in intracellular pools [207–209]. Recent studies have reported that chronic activity deprivation increases GRIP1 abundance at excitatory synapses and its interaction with GluA2, leading to synaptic targeting of AMPARs in cortical cultured neurons [76, 77]. In contrast, chronic enhancement of neuronal activity removes GRIP1 from excitatory synapses, which decreases surface AMPARs at synapses [76]. Compared to bidirectional modulation of synaptic GRIP1 expression in homeostatic synaptic plasticity, PICK1 expression is only altered by chronic activity blockade [78]. The TTX-induced synaptic scaling accompanies lysosome-mediated PICK1 degradation and can be occluded by genetic knock-out or shRNA knock-down of PICK1 [78]. Interestingly, pathologic levels of A*β* oligomers fail to reduce surface GluA2 expression and excitatory synaptic transmission in PICK knock-out neurons [79], indicating that GluA2 interaction with PICK1 mediates A*β*-induced synaptic depression. Thus, A*β*-dependent modulation of PICK1 and GRIP1 levels may likely contribute to aberrant developmental metaplasticity in AD.

AMPARs at excitatory synapses are also regulated by scaffolding proteins of the membrane associated guanylate kinase (MAGUK) family, which includes PSD-95, PSD-93, and SAP102 [210]. Chronic activity blockade increases synaptic accumulation of PSD95 and SAP102, whereas chronic activity enhancement decreases synaptic accumulation of PSD95 alone [84–86]. Double knock-down of PSD95/PSD93 or triple knock-down of PSD95/PSD93/SAP102 completely blocks chronic inactivity-induced increase in mEPSC amplitude [84], suggesting that PSD95 and PSD93 mediate synaptic scaling. In contrast, synaptic downscaling requires the PDZ1/2 domains of PSD-95 [84], which interact with transmembrane AMPAR regulatory proteins [TARPs] [211, 212]. Since TARPs link PSD-95 to AMPARs and promote synaptic insertion and stabilization of AMPARs [211, 212], these findings raise the possibility that reduced PSD95-TARP interaction may contribute to synaptic downscaling. Importantly, decreased PSD-95 expression is evident in AD mouse models [87] and A*β* application in cortical neuronal culture leads to downregulation of PSD-95 expression and dispersal of Shank1 [88, 89], another scaffolding protein enriched in excitatory glutamatergic synapses [213]. Interestingly, synaptic accumulation of guanylate kinase-associated protein (GKAP), which links Shank1 to PSD95 [214, 215], is increased upon chronic inhibition of neural activity and decreased by chronic excitation [90]. Such regulation of synaptic GKAP targeting contributes to bidirectional homeostatic scaling of excitatory synaptic strength [90]. Consistent with reports that pathological levels of A*β* increase degradation of PSD-95 and GKAP [89, 91], diminished interactions between PSD-95, TARP, and GKAP could dysregulate homeostatic synaptic plasticity in AD.

### 4.2. AMPAR Trafficking Regulators

Multiple proteins regulate synaptic AMPAR density by controlling their trafficking. One of them is Arc/Arg3.1, which is an immediate early gene. Arc/Arg3.1 mRNAs accumulate at excitatory synapses, where they are locally translated following synaptic simulation [216, 217]. Arc/Arg3.1 protein facilitates AMPAR internalization from the postsynaptic membrane by interacting with endocytosis mediators, endophilin2/3 and dynamin [218]. Chronic activity blockade of hippocampal or cortical cultured neurons has been shown to decrease mRNA and protein expression of Arc/Arg3.1 [80, 81]. Further, genetic ablation of Arc/Arg3.1 increases basal mEPSC amplitude and surface density of GluA1 and occludes TTX-induced increase in synaptic scaling [80]. Conversely, chronic elevation of neuronal activity increases Arc/Arg3.1 levels and decreases surface density of GluA1, whereas this regulation is absent in Arc/Arg3.1 knock-out neurons [80]. In addition to the critical roles of Arc/Arg3.1 in homeostatic synaptic plasticity, Arc/Arg3.1 expression is elevated in the medial prefrontal cortex of human AD patients [82], suggesting that elevated Arc/Arg3.1 expression may likely lead to AMPAR internalization during AD pathogenesis. In support of this notion, Arc/Arg3.1 is required for metabotropic glutamate receptor- (mGluR-) dependent LTD [219], a form of LTD that is also induced by application of A*β* oligomers [33]. Furthermore, Arc/Arg3.1 has been shown to mediate activity-dependent generation of A*β* by binding to presinilin-1 and regulating *γ*-secretase trafficking [82]. Based on these reports, persistent elevated Arc/Arg3.1 expression may act in multiple ways to disrupt synaptic homeostasis in AD by enhancing A*β* production and reducing synaptic AMPAR density.

Another immediate early gene, Homer1a, also contributes to homeostatic synaptic plasticity in Arc/Arg3.1-independent pathway [83]. Homer1a interrupts crosslinking action of constitutively expressed forms of Homer [220], thereby activating group I mGluRs in the absence of glutamate [221]. Chronic elevation of activity enhances Homer1a mRNA and protein expression, whereas chronic inactivity reduces Homer1a expression in cortical cultured neurons [83]. Importantly, mGluR inhibition or genetic ablation of Homer1a blocks bidirectional scaling of mEPSC amplitude and surface AMPAR density [83], implicating mGluR signaling and Homer1a in homeostatic synaptic plasticity. Interestingly, elevated tyrosine phosphorylation of GluA2 has been observed in Homer1a knock-out neurons [83] whereas tyrosine phosphorylation of GluA2 is decreased following group 1 mGluR stimulation through striatal enriched protein phosphatase (STEP_61_) [95]. Although the specific Tyr residues on GluA2 regulated by STEP_61_ are unknown, the downregulation of GluA2 tyrosine phosphorylation decreases surface expression of GluA2-containing AMPARs [222, 223]. Our recent study has demonstrated that chronic activity deprivation decreases protein and mRNA expression of STEP_61_ and increases Tyr-phosphorylation of its substrates, including the NMDAR subunit GluN2B and the AMPAR subunit GluA2 in hippocampal cultured neurons [94]. Increasing STEP_61_ activity blocks the increases in mEPSC amplitude and Tyr-phosphorylation of GluN2B and GluA2 induced by chronic activity blockade [94], suggesting that downregulation of STEP_61_ is crucial for mediating homeostatic synaptic scaling. Conversely, chronic activity enhancement increases STEP_61_ expression and decreases Tyr-phosphorylation of GluN2B and GluA2 [94]. Interestingly, elevated STEP_61_ expression is observed in cortices of human AD patients and causes dephosphorylation and internalization of AMPARs in AD mouse models [95–97]. Further, genetic ablation or pharmacologic inhibition of STEP_61_ prevents cognitive deficits and impaired hippocampal LTP in AD mouse models [96–98]. Given that STEP_61_ may also participate in metaplasticity [224], persistent elevation of STEP_61_ and Homer1a may disrupt developmental metaplasticity in AD.

In addition, alterations in Ca^2+^ influx modulates Ca^2+^-dependent activation of kinases such as Polo-like kinase 2 (Plk2) and Cyclin D kinase 5 (Cdk5) as well as protein phosphatases including calcineurin and protein phosphatase-1 (PP1) during homeostatic synaptic plasticity [93, 144, 145]. The increases in Plk2 and Cdk5 activity are thought to contribute to synaptic downscaling [144, 145] and AD pathogenesis [146]. Calcineurin-induced dephosphorylation of GluA1 at Ser845 has also been implicated in homeostatic synaptic plasticity [92] and AD [93]. Since PP1 activity downstream of calcineurin stimulation is required for LTD [225, 226], calcineurin may contribute to metaplasticity by regulating phosphorylation status of proteins which alters synaptic AMPAR density and function. In addition, Ca^2+^ influx through L-type voltage-gated Ca^2+^ channels (VGCCs) has been shown to increase PP1 activity via Ser43-phosphorylation of PP1 inhibitor-2 (I-2) following chronic activity elevation in hippocampal cultured neurons [99]. Furthermore, selective inhibition of PP1 blocks downscaling of surface AMPAR expression and mEPSC amplitude induced by chronic activity [99] as well as A*β*-induced impairment in hippocampal LTP [100], providing PP1 as another candidate signaling protein that may contribute to aberrant metaplasticity in AD.

### 4.3. Posttranslational Modification of AMPAR

Recent studies have revealed posttranslational modifications in addition to phosphorylation as important regulatory mechanisms of AMPAR expression during homeostatic synaptic plasticity. One such modification is palmitoylation, which mediates covalent attachment of palmitic acid [227]. The TTX-induced chronic silencing of network activity causes palmitoylation enzyme DHHC2 to be translocated from the dendrite to the postsynaptic density, resulting in homeostatic accumulation of PSD-95 and AMPARs at excitatory synapses [86]. Given that AMPAR trafficking is dynamically regulated by subunit-selective palmitoylation [228–230], these studies implicate palmitoylation of AMPAR subunits in the mechanism of synaptic scaling. Synaptic scaling also involves SUMOylation [103], which mediates covalent attachment of small ubiquitin-like modifiers (SUMO) [231]. Although there is no direct evidence for SUMOylation of AMPAR subunits [232], the TTX-induced elevation of surface AMPAR expression requires SUMOylation of Arc/Arg3.1 [103], a known regulator of AMPAR endocytosis [218]. Interestingly, reduced SUMOylation is observed in adult AD model mice [104]. While inhibition of SUMOylation blocks hippocampal LTP and hippocampal-dependent learning and memory in wild-type mice, the upregulation of SUMOylation by supplying its conjugating enzyme, Ubc9, rescues A*β*-induced deficits in hippocampal LTP and learning and memory [104]. Hence, reduced SUMOylation of Arc/Arg3.1 may contribute to defective developmental metaplasticity in AD.

Lastly, AMPARs are subjected to activity-dependent ubiquitination by the E3 ubiquitin ligase Nedd4-1, leading to their internalization and degradation in lysosomes [233–235]. Chronic elevation of neuronal activity increases Nedd4-1 protein levels, whereas shRNA-mediated knock-down of Nedd4-1 blocks the homeostatic reduction of surface AMPAR expression and mEPSC amplitudes induced by chronic activity [101], indicating that Nedd4-1 is required for homeostatic downscaling of excitatory synaptic strength. Given that elevated Nedd4-1 expression is found in human AD brain tissues [102], dysregulation of Nedd4-1 levels and subsequent impairment in homeostatic synaptic plasticity may play a role in AD etiology.

### 4.4. Regulation of Transcription and Translation

Ca^2+^ influx through NMDARs or L-type VGCCs activates signaling pathways that regulate transcriptions of genes important for neural development and plasticity. Consistent with this assertion, downscaling of excitatory synaptic strength induced by prolonged excitation of hippocampal CA1 neurons requires Ca^2+^ influx through L-type VGCCs and transcription activated downstream of CaMKK/CaMK4 signaling pathways [139]. TTX-induced synaptic scaling also requires transcription and translation; however, the mechanism involves a decrease in somatic Ca^2+^ influx through L-type VGCCs and subsequent reduction in CaMKK/CaMK4 signaling pathways [138, 140]. Recently, chronic inactivity was shown to increase transcription of genes encoding AMPARs and proteins that regulate AMPAR trafficking by decreasing cytosine methylation of genes [236]. Consistently, inhibition of DNA methylation alone induces synaptic scaling [236]. Furthermore, loss of methyl-CpG-binding protein-2 (MeCP2) prevents synaptic scaling in the visual cortex upon visual deprivation* in vivo* [143]. Taken together, these studies suggest that bidirectional homeostatic synaptic plasticity involves epigenetic modulation of genes whose protein products regulate excitatory synaptic transmission.

Our laboratory recently identified genes regulated by chronic alterations of neuronal activity in hippocampal neurons using unbiased gene expression analysis [81]. We identified several immediate early genes as well as genes associated with gene ontology terms “synaptic transmission” and “regulation of synaptic plasticity” [81]. One of the immediate early genes encodes brain-derived neurotrophic factor (BDNF) [81]. BDNF, which is secreted in an activity-dependent manner [237], regulates synaptic transmission and plasticity and promotes neuronal survival and transcription [238, 239]. We showed that BDNF mRNA expression is decreased in cultured hippocampal neurons upon chronic activity blockade using TTX treatment [81]. Importantly, inhibition of TrkB receptor signaling alone causes synaptic scaling in a similar extent as prolonged TTX treatment [115] whereas exogenous BDNF application prevents TTX-induced synaptic scaling [115] presumably through activation of mitogen- and stress-activated kinase 1 (MSK1) [141]. Interestingly, downregulation of BDNF is associated with the degree of synaptic and cognitive deficits during AD progression [116, 117, 240] and MSK1 activity is also elevated in AD [142]. These studies raise a possibility that aberrant BDNF-TrkB-MSK1 signaling pathway may disrupt synaptic homeostasis in AD.

In addition to the importance of transcriptional regulation, dendritic protein synthesis may serve as a mechanism to locally maintain the stability of synaptic strength. Chronic silencing of excitatory synaptic inputs stimulates dendritic protein synthesis by increasing the activity of eukaryotic elongation factor-2 (eEF2) [105]. Furthermore, simultaneous treatment of hippocampal neurons with TTX (to block action potentials) and APV (to block NMDAR-mediated miniature synaptic transmission) increases the expression of GluA1 homomers by stimulating local dendritic translation of GluA1 mRNAs [107, 241–243]. This synaptic scaling is mediated by microRNA-92a, which is a small noncoding RNA that inhibits translation of GluA1 mRNAs by binding to their 3′ untranslated region (UTR) [106]. Other studies have also reported that retinoic acid (RA) signaling via RA receptor-*α* (RAR*α*) interaction with the 5′ UTR of GluA1 mRNA contributes to synaptic scaling following prolonged cotreatment with TTX and APV by stimulating local dendritic synthesis of GluA1 [107–109]. Importantly, RAR signaling has been shown to regulate the expression of genes related to APP processing [110–113], attenuate A*β* deposition, and rescue memory deficits in AD mouse models [114], suggesting that alteration of RA signaling pathways may contribute to impaired metaplasticity in AD.

### 4.5. Cell Adhesion Molecules (CAMs)

*β*3 integrin is a cell adhesion molecule (CAM) enriched in excitatory synapses [244, 245] and controls synaptic currents mediated by GluA2-containing AMPARs [128]. Synaptic scaling induced by chronic activity blockade is associated with enhanced surface expression of *β*3 integrin in hippocampal neurons and is absent in *β*3 integrin knock-out neurons [128, 129]. Pharmacological perturbation of *β*3 integrin enhances GluA2 internalization and reduces synaptic AMPAR currents by activating the small GTPase Rap1 [128], which has been implicated in homeostatic downscaling of excitatory synapses [144, 246]. In addition to *β*3 integrin, class I major histocompatibility complex (MHC-1) proteins, which are found postsynaptically at excitatory synapses, also contribute to synaptic scaling following chronic activity blockade [130]. Although the role of *β*3 integrin and MHC-1 in AD pathogenesis remains unknown, their neuronal expression is regulated by glia-derived tumor necrosis factor *α* (TNF*α*) [128, 247, 248], which is involved in AD pathology in humans and AD mouse models [118–124]. TNF*α* elevates AMPAR-mediated mEPSC amplitude through activation of TNF*α* receptor during synaptic scaling [125, 126] whereas TNF*α* knock-out mice lack synaptic scaling in their visual cortex [127] but display normal LTP [127, 249, 250]. Hence, TNF*α* may influence metaplasticity through *β*3 integrin and MHC-1, and such a signaling pathway may be disrupted in AD.

N-Cadherin is another CAM that is enriched at excitatory synapses and has been implicated in AD as well as homeostatic synaptic plasticity. N-Cadherin promotes APP dimerization, modulates A*β* secretion, and reduces surface expression of presinilin-1 [131, 132]. N-Cadherin also binds to the extracellular domains of GluA1 in a Ca^2+^-dependent manner and regulates GluA1 surface expression [251, 252]. Although N-Cadherin interaction with the actin cytoskeleton [133, 253] contributes to dendritic spine enlargement during LTP expression [253–257], the interaction between N-Cadherin and *β*-catenin mediates bidirectional homeostatic synaptic plasticity by regulating GluA1-containing AMPARs [133, 134]. Given that inhibition of N-Cadherin interaction with *β*-catenin accelerates A*β*-induced synaptic impairments [135], dysregulation of N-Cadherin may likely impair homeostatic synaptic plasticity in AD. Ephrin receptor tyrosine kinase subfamily EphA4 is another CAM implicated in homeostatic synaptic plasticity. Increased activity of EphA4 mediates homeostatic downscaling by stimulating ubiquitin-dependent proteasome degradation of GluA1 [136]. Interestingly, soluble A*β* oligomers induce EphA4 activation, whereas genetic ablation or inhibition of EphA4 prevents hippocampal LTP impairment in AD transgenic model mice [137], raising an interesting possibility that A*β*-induced enhancement in EphA4 activity may impair metaplasticity in AD by regulating AMPAR degradation.

## 5. Conclusions

Recent studies have uncovered an exciting link between pathologic accumulation of A*β* and aberrant metaplasticity, a form of homeostatic synaptic plasticity that controls the induction threshold for LTP and LTD. Specifically, these studies have suggested a novel hypothesis that aberrant metaplasticity may contribute to LTP inhibition and LTD enhancement in AD. However, the molecular mechanism underlying A*β*-dependent alteration of metaplasticity remains largely unknown. Since many molecular players involved in homeostatic synaptic plasticity have been shown to regulate synaptic AMPAR density in Hebbian synaptic plasticity, it is tempting to speculate that pathologic levels of A*β* mediate their effect via a common mechanism shared between Hebbian and homeostatic plasticity at excitatory synapses. Challenges lie ahead in understanding how the molecular players and pathways reviewed here work together to express homeostatic plasticity at excitatory synapses and how A*β* disrupts homeostatic synaptic plasticity in AD. Future studies designed to tackle these challenges should offer substantial insights into the homeostatic control of excitatory synaptic strength in normal brain and AD brain. These studies may also facilitate the search for targeted therapeutic interventions to correct aberrant metaplasticity in AD, thus reversing persistent synaptic weakening and cognitive dysfunction in AD.

## Figures and Tables

**Table 1:** Molecular mechanisms and players involved in AD and the expression of homeostatic synaptic plasticity.

	Roles in synaptic scaling	Involvement in AD	References
AMPAR scaffolding proteins			
GRIP1	Synaptic accumulation and removal of GRIP1 mediate synaptic scaling and downscaling, respectively, by regulating synaptic AMPAR targeting.		[76, 77]
PICK1	PICK1 degradation mediates synaptic scaling.	PICK1 interaction with GluA2 mediates A*β*-induced synaptic depression.	[78, 79]

Regulators of AMPAR trafficking			
Arc/Arg3.1	Downregulation of Arc/Arg3.1 mediates synaptic scaling by increasing surface AMPAR density. Upregulation of Arc/Arg3.1 mediates synaptic downscaling by promoting AMPAR endocytosis.	Arc/Arg3.1 expression is elevated in AD and mediates activity-dependent generation of A*β* by binding to presinilin-1 and regulating *γ*-secretase trafficking.	[80–82]
Homer1a	Downregulation of Homer1a mediates synaptic scaling, whereas upregulation of Homer1a mediates synaptic downscaling by regulating surface AMPAR density and Tyr-phosphorylation.		[83]

Regulators of synaptic AMPAR density			
PSD-95	Synaptic accumulation of PSD-95 mediates synaptic scaling, whereas its interaction with TARP mediates synaptic downscaling.	Pathological level of A*β* leads to PSD-95 degradation.	[84–89]
PSD-93	PSD-93 mediates synapticscaling.		[84]
GKAP	Synaptic accumulation and removal of GKAP mediate synaptic scaling and downscaling, respectively, by regulating surface AMPAR density.	Pathological level of A*β* leads to GKAP degradation.	[90, 91]

Posttranslation modification of AMPAR			
Calcineurin	Reduced calcineurin activity mediates synaptic scaling via GluA1-Ser845 dephosphorylation and subsequent synaptic trafficking of Ca^2+^-permeable AMPARs.	In AD mouse model, increased activity of calcineurin induces dephosphorylation and synaptic removal of the GluR1 subunit of AMPAR.	[92, 93]
STEP_61_	Downregulation of STEP_61_ mediates synaptic scaling, whereas enhanced STEP_61_ upon chronic activity induces dephosphorylation of GluN2B and GluA2.	STEP_61_ expression is elevated in AD and mediates A*β*-induced dephosphorylation and internalization of NMDARs and AMPARs, whereas inhibition of STEP_61_ prevents cognitive deficits and impaired hippocampal LTP in AD mouse models.	[94–98]
PP1	Downregulation of PP1 inhibitor-2 (I-2) mediates synaptic downscaling by reducing surface AMPARs.	Inhibition of PP1 blocks A*β*-induced impairment in hippocampal LTP.	[99, 100]
DHHC2	Translocation of DHHC2 to PSD mediates synaptic scaling by enhancing synaptic targeting of PSD95 and AMPAR.		[86]
Nedd4-1	Upregulation of Nedd4-1 mediates synaptic downscaling by reducing surface AMPAR density.	Nedd4-1 expression is elevated in AD.	[101, 102]
SUMO-1 and Ubc9	SUMOylation of Arc/Arg3.1 mediates synaptic scaling.	SUMO-conjugating enzyme, Ubc9, enhances SUMOylation and rescues A*β*-induced deficits in hippocampal LTP and learning and memory.	[103, 104]

Local dendritic translation of AMPAR			
eEF2	Increased eEF2 activity mediates synaptic scaling by stimulating local dendritic synthesis.		[105]
miRNA-92a	Inhibition of miRNA-92A mediates synaptic scaling by stimulating local dendritic synthesis of GluA1.		[106]
Retinoic acid (RA)	Increased RA activity mediates synaptic scaling by stimulating local dendritic synthesis of GluA1 through RA receptor.	RA regulates the expression of APP processing genes, attenuates A*β* deposition, and rescues memory deficits in AD mouse model.	[107–114]

Secreted factors			
BDNF	Downregulation of BDNF mediates synaptic scaling.	Downregulation of BDNF levels is associated with the degree of synaptic and cognitive deficits during the progression of AD.	[81, 115–117]
TNF*α*	TNF*α* mediates synaptic scaling in primary neuronal culture and visual cortex upon activity deprivation.	TNF*α* contributes to AD-related brain neuroinflammation and amyloidogenesis via *β*-secretase regulation.	[118–127]

Cell adhesion molecules			
*β*3 integrin	Enhanced surface expression of *β*3 integrin inhibits the small GTPase Rap1 and mediates synaptic scaling by stabilizing synaptic.		[128, 129]
MHC-1	MHC-1 mediates TTX-induced synaptic scaling in hippocampal cultured neurons.		[130]
N-Cadherin	N-Cadherin interaction with *β*-catenin mediates synaptic scaling and downscaling by regulating GluA1-containing AMPARs.	Inhibition of N-Cadherin interaction with *β*-catenin accelerates A*β*-induced synaptic impairments.	[131–135]
EphA4	Increased Eph4 activity mediates synaptic downscaling by stimulating ubiquitin-dependent proteasome degradation of GluA1.	Soluble A*β* oligomers upregulate EphA4 whereas genetic ablation or inhibition of EphA4 prevents hippocampal LTP impairment in AD transgenic model mice.	[136, 137]

Transcriptional regulation			
CaMKK-CaMK4	Reduced activity of the CaMKK/CaMK4 signaling pathway mediates synaptic scaling, whereas its stimulation mediates synaptic downscaling.		[138–140]
MSK1	MSK1 mediates TTX-induced synaptic scaling in hippocampal neurons by increasing surface AMPAR density.	MSK1 activity is elevated in AD.	[141, 142]
MeCP2	MeCP2 mediates synaptic scaling in visual cortex upon visual deprivation *in vivo*.		[143]

Other proteins			
Plk2	Increase in Plk2 activity mediates synaptic downscaling.		[144, 145]
Cdk5	Increase in Cdk5 activity mediates synaptic downscaling.	Enhanced Cdk5 activity in AD contributes to Tau phosphorylation and toxicity.	[144, 146]
